# Extracellular vesicles in stem cell-based gonadal regeneration: mechanisms, therapeutic potential, and translational challenges

**DOI:** 10.3389/fvets.2025.1645266

**Published:** 2026-04-20

**Authors:** Rodrigo Paolo Flores Abuna, Lucas Fornari Laurindo, Sandra Maria Barbalho, Maria Angélica Miglino

**Affiliations:** 1 Department of Animal Anatomy, School of Veterinary Medicine, Universidade de Marília (UNIMAR), Marília, Brazil; 2 Division of Cellular Growth, Hemodynamic, and Homeostasis Disorders, Faculdade de Medicina, Universidade de São Paulo (USP), São Paulo, Brazil; 3 School of Medicine, Universidade de Marília (UNIMAR), Marília, Brazil; 4 Regenerative Medicine Laboratory “Carlos Augusto Camargo de Souza Baptista”, Universidade de Marília (UNIMAR), Marília, Brazil

**Keywords:** extracellular vesicles, gonads, regeneration, regenerative medicine, stem cell therapy

## Abstract

Gonadal dysfunction resulting from conditions such as premature ovarian insufficiency, chemotherapy-induced damage, or genetic disorders often leads to infertility and hormone imbalance. Although assisted reproductive technologies and hormone replacement therapies mitigate clinical symptoms, they remain incapable of reinstating native gonadal architecture and physiological function. In recent years, stem cell-based therapies, particularly those employing mesenchymal stem cells (MSCs), have demonstrated regenerative potential. However, limitations including poor engraftment, potential tumorigenicity, and ethical concerns, have accelerated the paradigm shift toward extracellular vesicles (EVs) as a safer, cell-free alternative. EVs derived from MSCs are membrane-bound nanovesicles enriched with regulatory microRNAs, proteins, and lipids that exert potent paracrine effects. These vesicles modulate apoptosis, inflammation, fibrosis, and angiogenesis. MSC-EVs can restore folliculogenesis, support spermatogenesis, and normalize hormonal profiles in preclinical models of ovarian and testicular failure. Notably, EVs derived from adipose tissue, bone marrow, placenta, or amniotic membrane exhibit regenerative potential while mitigating the risks associated with live-cell transplantation. This review synthesizes current advances in MSC-EV-based therapies for gonadal regeneration, highlighting their integration into reproductive tissue engineering. Incorporating EVs into decellularized extracellular matrix scaffolds offers promising strategies for targeted tissue repair, extending their application in organoid systems for *in vitro* gametogenesis, disease modeling, and drug screening. Despite challenges related to EV heterogeneity, standardization of isolation, and delivery strategies, MSCs-derived EVs represent a transformative and ethically sound platform for restoring fertility and endocrine function.

## 1 Introduction: the need for regenerative solutions in reproductive medicine

The gonads, specifically the testes in males and the ovaries in females, are essential for both reproductive and endocrine functions. They regulate fertility, sex hormone production, and systemic homeostasis by interacting with the hypothalamic–pituitary-gonadal (HPG) axis. In females, folliculogenesis is a tightly controlled, multistage process governed by follicle-stimulating hormone (FSH), luteinizing hormone (LH) (1), and a network of paracrine signals including growth differentiation factor 9 (GDF9), bone morphogenetic proteins (BMPs), and signaling cascades such as Notch, Wnt, and Hedgehog. These pathways collectively orchestrate granulosa cell (GC) proliferation, follicular maturation, and ovulation (2, 3). Following ovulation, the remaining follicular cells differentiate into the corpus luteum, a temporary endocrine structure that secretes progesterone to maintain the luteal phase and prepare the endometrium for possible implantation (3, 4).

In males, spermatogenesis occurs in the seminiferous tubules and relies on testosterone-secreting Leydig cells, which LH activates. Sertoli cells, stimulated by FSH, support the development of germ cells. These processes are tightly regulated by the HPG axis, culminating in the production of spermatozoa from spermatogonial stem cells (SSC) (5–7). Despite this tight regulation, gonadal function is highly vulnerable to both internal and environmental stressors, including oxidative damage, endocrine-disrupting chemicals, and nutritional deficits such as vitamin D insufficiency (8, 9). For instance, in chronic psychological stress, elevated cortisol levels impair gonadotropin-releasing hormone (GnRH) secretion (9–13), leading to reduced FSH and LH activity, thereby compromising gametogenesis and hormone balance (10, 14).

Gonadal dysfunction can result from diverse etiologies, including chemotherapeutic agents (15), genetic disorders like the Turner and Klinefelter syndromes, autoimmune diseases, and physiological aging (16). These conditions may culminate in premature ovarian insufficiency (POI), azoospermia, or hypogonadism, significantly impairing fertility and quality of life (17). Although assisted reproductive technologies (ART) and hormone replacement therapy have transformed fertility care, they primarily manage symptoms without restoring native gonadal structure or function. Furthermore, ART success rates decline with age and repeated cycles, while hormone therapies carry risks and often fail to reconstitute endogenous endocrine feedback loops (18–22).

Gonadal tissue transplantation has also been explored as an approach to restore fertility. However, challenges such as ischemic injury, immunosuppression requirements, and the potential reintroduction of malignant cells, especially in cancer survivors, have limited its clinical application (23). Similarly, SSC transplantation and *in vitro* gametogenesis (IVG) using pluripotent stem cells (PSCs) offer experimental avenues for gamete generation but remain under investigation due to concerns about safety and functionality (24–28).

Stem cell-based therapies, IVG, and tissue engineering have shown potential in restoring gonadal architecture and function. Mesenchymal stem cells (MSCs) derived from bone marrow (BM-MSCs), adipose tissue (AT-MSCs), umbilical cord (UC-MSCs), or placenta (Pc-MSCs) have demonstrated regenerative effects in preclinical models. Mechanisms of action involve paracrine signaling, angiogenesis, immunomodulation, and anti-apoptotic activity (29–34).

Even though stem cell-based therapies are promising, growing evidence suggests that extracellular vesicles (EVs) derived from MSCs may replicate many therapeutic benefits of stem cells while avoiding the risks associated with live-cell transplantation (35–38). EVs are nanoscale membrane-bound vesicles enriched in bioactive molecules, including microRNAs (miRNAs/miR), proteins, and lipids, which can modulate the behavior of recipient cells (39, 40). Their ability to inhibit apoptosis, promote angiogenesis, modulate immune responses, and restore follicular development expanded the paradigm toward cell-free tools in reproductive regenerative medicine (35, 41, 42).

This review focuses on the role of MSCs-derived EVs in gonadal regeneration, emphasizing female reproductive restoration. We critically explore their mechanism of action, therapeutic potential in ovarian and testicular failure, integration with biomaterials and tissue engineering platforms, and current translational challenges. By synthesizing preclinical and emerging clinical evidence, we aim to highlight EVs as next-generation agents for cell-free regenerative therapy in reproductive medicine.

## 2 Literature search

### 2.1 Databases and timeframe

A targeted literature search was conducted using PubMed, Scopus, and Web of Science to identify studies relevant to MSCs-derived EVs in gonadal regeneration. The search included publications up to November 2025.

### 2.2 Search strategy

The search employed combinations of terms including “mesenchymal stem cells,” “MSCs-derived extracellular vesicles,” “exosomes,” “gonadal regeneration,” “ovarian failure,” “testicular failure,” “folliculogenesis,” “spermatogenesis,” and “reproductive tissue engineering.”

### 2.3 Inclusion and exclusion criteria

Studies were included if they were original research, preclinical investigations, or emerging clinical studies examining MSCs-derived EVs-mediated ovarian or testicular regeneration, and if they provided mechanistic insights or explored therapeutic applications. Review articles and seminal works were incorporated selectively to provide context and support critical discussion. Studies were excluded if they were unrelated to gonadal regeneration or lacked sufficient methodological detail.

### 2.4 Selection and approach

Relevant studies were selected based on relevance, methodological rigor, and contribution to understanding the mechanisms, therapeutic potential, biomaterial integration, and translational challenges of MSCs-derived EVs. This targeted narrative approach enabled a focused and critical synthesis of current knowledge, highlighting MSCs-derived EVs as promising cell-free agents for reproductive tissue regeneration.

## 3 Mesenchymal stem cells in gonadal regeneration

Although the present review focuses primarily on MSCs-derived products, a brief characterization of MSC surface markers is essential because the phenotypic profile of the parental cells directly influences the composition, potency, and regenerative properties of their secretome and EVs. MSCs are multipotent stromal cells that can differentiate into various mesodermal lineages and exert regenerative effects through paracrine signaling. Their therapeutic potential in gonadal regeneration, particularly for female infertility, has been widely demonstrated in preclinical studies (26, 43). The International Society for Cellular Therapy (ISCT) defines MSCs by their adherence to plastic, expression of the surface antigens CD73, CD90, CD105, CD44, and Scal-1, and lack of hematopoietic markers such as CD45, CD14, CD19, CD34, CD79𝛼, CD11b, and HLA-DR. They must also be able to differentiate into adipogenic, osteogenic, and chondrogenic lineages (44, 45). However, each host tissue has a specific combination of marker expression (46). For instance, pluripotency-associated markers such as Oct-4, Nanog, Rex-11, SSEA-3, SSEA-4, Tra-1-60, and Tra-1-81 have been detected in MSCs isolated from human fetal blood, liver, and bone marrow during the first trimester. They are not typically found in cultured MSCs derived from adult bone marrow (47).

Soncini et al. described a similar expression profile in MSCs isolated and analyzed from bone marrow, amniotic membrane, and chorionic membrane (48). CD271 is associated with stemness, clonogenic potential, and tissue tropism, which may have an impact on stronger tissue-repair signaling, and its reduction during passaging or expansion could lead to a decrease in the therapeutic efficacy of MSCs-derived EVs (49).

MSCs can be isolated from various tissues, including bone marrow, adipose tissue, umbilical cord, menstrual blood, endometrium, and placenta. In models of gonadal injury, MSCs have demonstrated the ability to migrate to damaged ovarian tissue and respond to local molecular cues (50, 51). While they were initially believed to contribute to regeneration via direct differentiation into GC or thecal cells, current evidence suggests that MSCs primarily exert their effects by modulating the ovarian microenvironment (50, 52, 53).

The therapeutic efficacy of MSCs largely depends on their secretome, which includes bioactive molecules such as insulin-like growth factor (IGF), vascular endothelial growth factor (VEGF), hepatocyte growth factor (HGF), and transforming growth factor-beta (TGF-*β*). These molecules promote angiogenesis, reduce oxidative stress, modulate inflammation, and enhance tissue survival and repair (54). Some regenerative effects are also attributed to their ability to modulate signaling pathways such as phosphoinositide 3-kinase/protein kinase B (PI3K/Akt), extracellular signal-regulated kinase (ERK) 1/2, and Wnt/*β*-catenin, which are involved in cell proliferation and survival (55–57).

Another important mechanism is mitochondrial transfer. Under inflammatory conditions, MSCs form tunneling nanotubes that deliver functional mitochondria to injured GCs and stromal cells, improving cellular viability and metabolic activity (58, 59). Cytokines such as interleukin (IL)-6 and tumor necrosis factor alpha (TNF-*α*) influence this process and may enhance ovarian function by rescuing damaged cells.

Furthermore, MSCs possess potent immunomodulatory properties. They regulate immune responses through cytokines such as IL-10, TGF-*β*, and prostaglandin E2 (PGE2), suppressing pro-inflammatory signaling and promoting tissue repair. This is especially relevant in autoimmune-associated gonadal dysfunctions and inflammatory conditions like polycystic ovary syndrome (PCOS) (60, 61).

**Figure 1:**
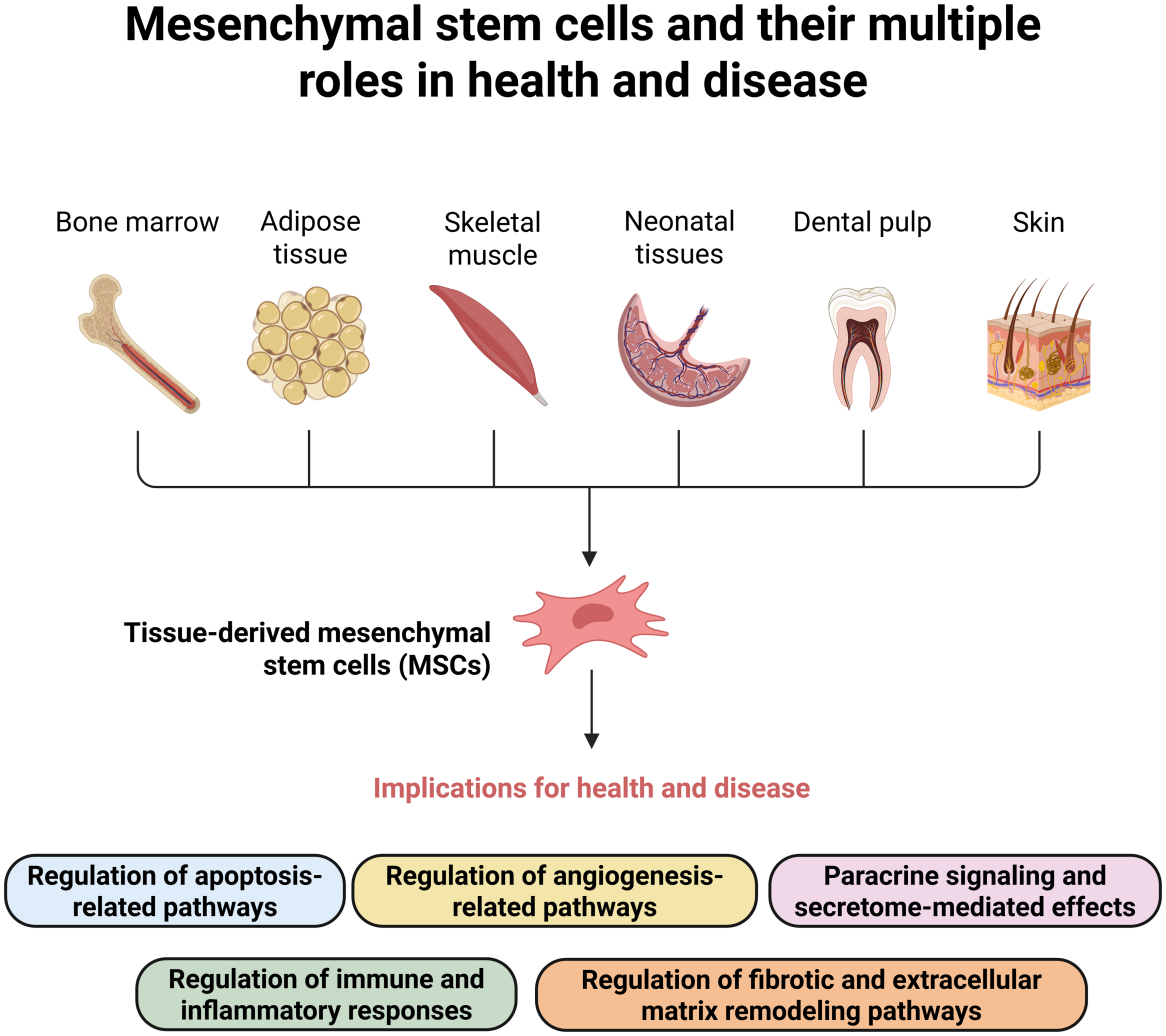
Sources of mesenchymal stem cells (MSCs) and their significant functional effects on health and disease. MSCs can be isolated from multiple tissues, including bone marrow, adipose tissue, skeletal muscle, neonatal tissues (e.g., umbilical cord), dental pulp, and skin. MSCs exert diverse biological functions through direct and paracrine mechanisms. Key processes regulated by MSCs include antiapoptotic signaling, promotion of angiogenesis, antifibrotic activity, immunomodulation, and broader paracrine effects that influence tissue repair and inflammation. Created with BioRender (https://BioRender.com).

Figure 1 illustrates the multiple roles of MSCs in body homeostasis, including their effects in immune modulation, angiogenesis, apoptosis, and fibrosis.

## 4 Mechanisms of action of MSCs-derived EVs for gonadal regeneration

**Figure 2:**
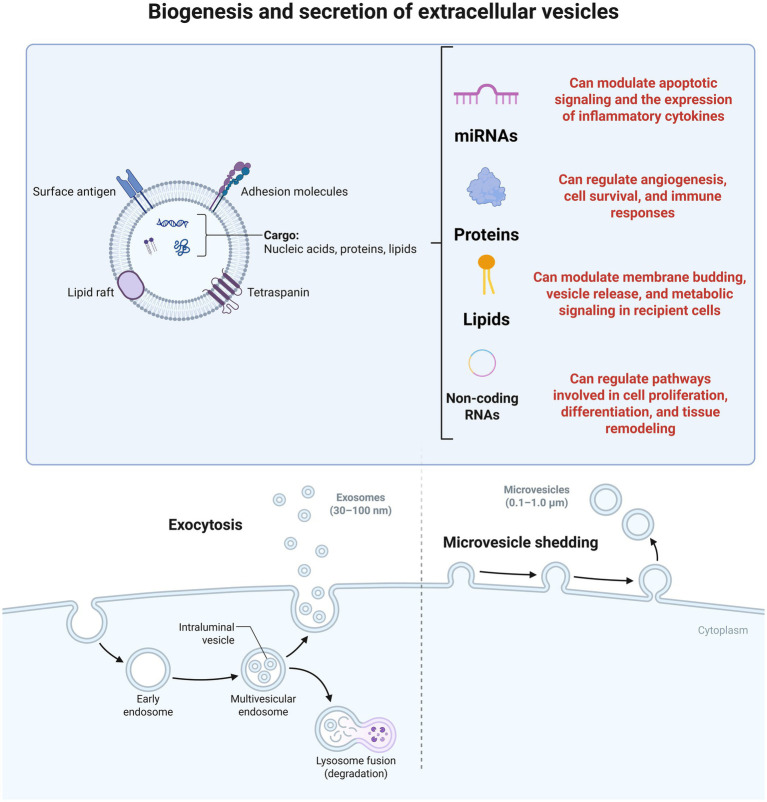
Biogenesis and molecular composition of extracellular vesicles (EVs). Extracellular vesicles encompass a heterogeneous population of membrane-bound particles that carry bioactive cargo, including nucleic acids, proteins, and lipids. The upper panel illustrates key structural features of EVs, such as surface antigens, adhesion molecules, lipid rafts, and tetraspanins, along with representative cargo categories: microRNAs (involved in protein inhibition), proteins (diverse functional roles), lipids (metabolic regulation), and non-coding RNAs (regulation of regeneration). The lower panel depicts the two main pathways of EV release. Exocytosis leads to the secretion of exosomes (30–100 nm), formed as intraluminal vesicles within multivesicular endosomes that either fuse with lysosomes for degradation or with the plasma membrane for release. Ectocytosis/microvesicle shedding generates microvesicles (0.1–1.0 μm) via outward budding and plasma membrane fission. Together, these pathways contribute to the dynamic production of EVs that mediate intercellular communication in health and disease. Created with BioRender (https://BioRender.com).

A set of biological mechanisms drives the therapeutic efficacy of MSCs-derived EVs in gonadal regeneration. These include modulation of angiogenesis, suppression of apoptosis, attenuation of fibrosis, and immunomodulatory effects. By delivering bioactive molecules, mainly miRNAs, EVs influence cell behavior in the ovarian and testicular microenvironments. Figure 2 depicts EV formation, including their cargo evaluation and possible effects on the body.

### 4.1 Angiogenic effects

Angiogenesis is essential for restoring gonadal function, ensuring nutrient supply, oxygenation, and hormonal exchange. MSCs-derived EVs enhance neovascularization through the delivery of pro-angiogenic molecules such as VEGF, stromal cell-derived factor 1 alpha (SDF-1𝛼), IGF-1, HGF, and basic fibroblast growth factor (bFGF) (62). VEGF plays a central role in ovarian follicle maturation, corpus luteum function, and testicular development. Upon interaction with its receptors, VEGF activates the PI3K/Akt and glycogen synthase kinase 3 beta (GSK3*β*)/β-Catenin signaling pathways, triggering endothelial cell proliferation, migration, and differentiation (56, 63). IGF-1, similarly, supports GCs’ survival and promotes angiogenesis via PI3K/Akt signaling (64). EVs enriched with miR-126-3p, known to activate the PI3K/Akt/endothelial nitric oxide synthase (eNOS) axis, further potentiate endothelial repair (65, 66).

Collectively, the cited studies indicate that MSCs-derived EVs promote angiogenesis by simultaneously activating multiple signaling pathways known to regulate endothelial cell survival and vascular remodeling. The roles of VEGF, IGF-1, and miR-126-3p described in these works consistently converge on PI3K/Akt-dependent pro-angiogenic mechanisms, aligning with reports that improved vascularization directly supports gonadal recovery. Thus, the literature collectively supports a model in which EV cargo reestablishes the vascular microenvironment required for functional tissue regeneration.

### 4.2 Anti-fibrotic properties

Fibrosis disrupts gonadal architecture and function by impairing folliculogenesis and hormonal secretion. MSCs-derived EVs counteract fibrogenesis by modulating the activity of TGF-β1, the main cytokine driving fibrotic responses. TGF-β1 signals through the SMAD pathway to promote extracellular matrix deposition and stroma thickening (67). Studies in POI models show that BM-MSCs or human UC-MSCs transplantation downregulates TGF-β1 expression, consequently reducing fibrosis (68). Additionally, EVs inhibit SMAD3 phosphorylation and regulate alternative antifibrotic cascades such as AMP-activated protein kinase (AMPK)/NR4A, ultimately leading to stromal cell reprogramming and improved follicle viability (69, 70).

Together, these findings demonstrate that MSC-based therapies exert anti-fibrotic effects by targeting both upstream mediators (such as TGF-β1 expression) and downstream signaling events (including SMAD3 phosphorylation). The cited literature emphasizes that EVs act through complementary mechanisms that ultimately reduce extracellular matrix accumulation and preserve the ovarian stromal environment. Consequently, EV-mediated modulation of fibrotic pathways forms a key component of their restorative potential in POI and related gonadal disorders.

### 4.3 Immunomodulatory effects

A chronic inflammatory setting often exacerbates gonadal dysfunction. MSCs-derived EVs reestablish immune homeostasis by inhibiting the production of pro-inflammatory cytokines, such as IL-1*β*, TNF-*α*, and interferon (IFN)-*γ*, while promoting the secretion of anti-inflammatory mediators like IL-10, TGF-β, and PGE2 (71). EVs suppress the activation of immune cells, including macrophages, dendritic cells, and T lymphocytes. Their immunoregulatory functions involve the delivery of enzymes like indoleamine 2,3-dioxygenase (IDO) and regulatory microRNAs that inhibit T cells and promote regulatory T cell (Treg) induction (72). Crawford et al. demonstrated that MSCs also drive the regeneration of functional Tregs, thus diminishing the inflammatory response (73).

The cited studies collectively show that MSCs-derived EVs modulate both the cytokine milieu and the activation state of key immune cell populations. By reducing pro-inflammatory signaling while enhancing Treg-mediated suppression, EVs help restore a tolerogenic environment that protects gonadal tissue from further inflammatory damage. The consistency of these findings across multiple models underscores the central role of immunomodulation in EV-mediated gonadal repair.

### 4.4 Anti-apoptotic effects

Apoptosis of GCs and germline cells underlies follicular depletion in POI and other gonadal disorders. MSCs-derived EVs suppress apoptosis through multiple signaling pathways. For example, EVs carrying miR-21, miR-644-5p, or miR-144-5p modulate apoptotic regulators such as PTEN, PDCD4 and p53, and increase anti-apoptotic Bcl2 expression (74, 75). These EVs also interfere with the apoptotic cascade by downregulating Caspase-3 and pro-apoptotic genes like Bax and p53 (42, 76). Moreover, EVs can inhibit endoplasmic reticulum stress-induced apoptosis in GCs by inhibiting the IRE1𝛂 endoplasmic reticulum signaling pathway in POI mouse models (77).

Overall, the cited literature demonstrates that MSCs-derived EVs counteract apoptosis by targeting PTEN, PDCD4, p53, and Bax, together with suppression of Caspase-3 activity, EVs act on multiple nodes of the apoptotic machinery. These convergent mechanisms explain the consistent observation of preserved GCs survival following EVs treatment in experimental models.

### 4.5 Paracrine signaling

MSCs act primarily via paracrine signaling, releasing cytokines and miRNAs that modulate inflammation and promote tissue repair (78). They secrete a variety of bioactive factors, including cytokines, growth factors, EVs, and miRNAs, which regulate cell survival, inflammation, promote angiogenesis, and modulate the immune system (79). One of the best-documented paracrine functions of MSCs is the promotion of neovascularization through the release of VEGF, HGF, and bFGF, which stimulate endothelial cell proliferation and enhance blood vessel formation (80). VEGF and IGF-1 also act to prevent GCs apoptosis and facilitate folliculogenesis (81–84). HGF is recognized for its robust anti-apoptotic properties in both GCs and oocytes, and supports neovascularization and ovarian functional recovery (85).

Their immunomodulatory properties, mediated by IL-10, TGF-*β*, and PGE2, suppress inflammatory responses and facilitate tissue healing (86). Additionally, MSCs-derived EVs and miRNAs play a critical role in intercellular communication, facilitating tissue remodeling, fibrosis reduction, and apoptosis inhibition (87).

In the context of reproductive medicine, MSCs-derived paracrine signals enhance the survival and function of reproductive cells by releasing IGF-1, leukemia inhibitory factor (LIF), and SDF-1, which in turn promote folliculogenesis, oocyte maturation, and spermatogenesis (42, 88, 89). In another study, brain-derived neurotrophic factor (BDNF), a neurotrophin secreted by BM-MSCs, enhances oocyte maturation and supports embryonic development (90). VEGF and bFGF are key mediators of ovarian angiogenesis, crucial for supplying nutrients to GCs and supporting follicle development.

These properties position MSCs as candidates for treating conditions such as POI, PCOS, and infertility. Khanmohammadi et al. demonstrated that BM-MSCs and their conditioned medium produced comparable outcomes in repairing ovarian damage (91). Similarly, BM-MSCs’ conditioned medium reduced apoptosis, promoted proliferation, and stimulated estrogen secretion (92). In another study, the conditioned medium of BM-MSCs demonstrated angiogenic potential by increasing the expression of angiogenesis-related genes, including TGF-*β* and CCL11, which promoted endothelial proliferation, neovascular formation, and enhanced capillary density. All these events were associated with the activation of the PI3K/Akt pathway (93).

However, not all studies support the equivalence of the conditioned medium and cell-based therapies. Ling et al. found that direct transplantation of MSCs has superior outcomes in reversing ovarian damage compared to the administration of conditioned medium alone (30). This suggests that while paracrine effects are essential, the complete therapeutic potential of MSCs may require direct cell-tissue interactions, from which new concerns arise, such as variability in MSCs sources, culture conditions, and differentiation efficiency that may impact reproducibility and clinical outcomes (94, 95).

In this context, MSCs act through a multifaceted network of regenerative mechanisms, including paracrine signaling, immunomodulation, angiogenesis, anti-apoptotic activity, and organelle transfer. While their therapeutic benefits are promising, increasing attention has turned to EVs as the key mediators of these effects, offering a safer and more controlled alternative to whole-cell therapy.

## 5 Extracellular vesicles: biogenesis and secretion

EVs are key mediators of cell-to-cell communication. Their composition reflects their cellular origin, and they are enclosed by a lipid bilayer. They lack the capacity for self-replication (44). EVs include surface receptors, membrane proteins, and diverse classes of ribonucleic acids such as messenger RNA, transfer RNA, ribosomal RNA, microRNA, small nucleolar RNA, and long non-coding RNAs (39). EVs were initially described as mechanisms for the clearance of unwanted cellular components (96).

EVs comprise a heterogeneous group of membrane-bound particles that mediate intercellular communication by transporting proteins, lipids, and nucleic acids (97). Classically, EVs are categorized by cellular origin and approximate size: exosomes (30–100 nm), microvesicles/ectosomes (0.1–1.0 μm), and apoptotic bodies (1–5 μm) (98).

Exosomes originate within the endolysosomal system. Their biogenesis begins with inward budding of the endosomal membrane to form intraluminal vesicles (ILVs) within multivesicular bodies (MVBs). MVB maturation and ILV formation may occur through endosomal sorting complex required for transport (ESCRT)-dependent mechanisms—involving ESCRT-0, -I, -II, -III complexes and associated proteins such as ALIX and VPS4—or through ESCRT-independent pathways relying on lipids such as ceramide, tetraspanins, and specific cargo-sorting mechanisms. MVBs can either fuse with lysosomes for degradation or with the plasma membrane to release ILVs as exosomes. Exosome secretion is regulated by small GTPases (e.g., Rab27a/b, Rab11, Rab35), SNARE proteins, and cytoskeletal dynamics (99, 100).

Microvesicles (ectosomes) are generated by outward budding and fission of the plasma membrane (101). This process is driven by changes in local membrane curvature, alterations in lipid asymmetry (e.g., phosphatidylserine externalization), and cytoskeletal rearrangements mediated by GTPases, actomyosin contractility, and calcium-dependent signaling (102).

Apoptotic bodies arise during programmed cell death (103). As cells undergo apoptosis, caspase activation leads to chromatin condensation, cell shrinkage, membrane blebbing, and eventual fragmentation into large vesicles (1–5 μm). These apoptotic bodies contain organelles, nuclear fragments, and cytoplasmic components (104, 105).

**Figure 3:**
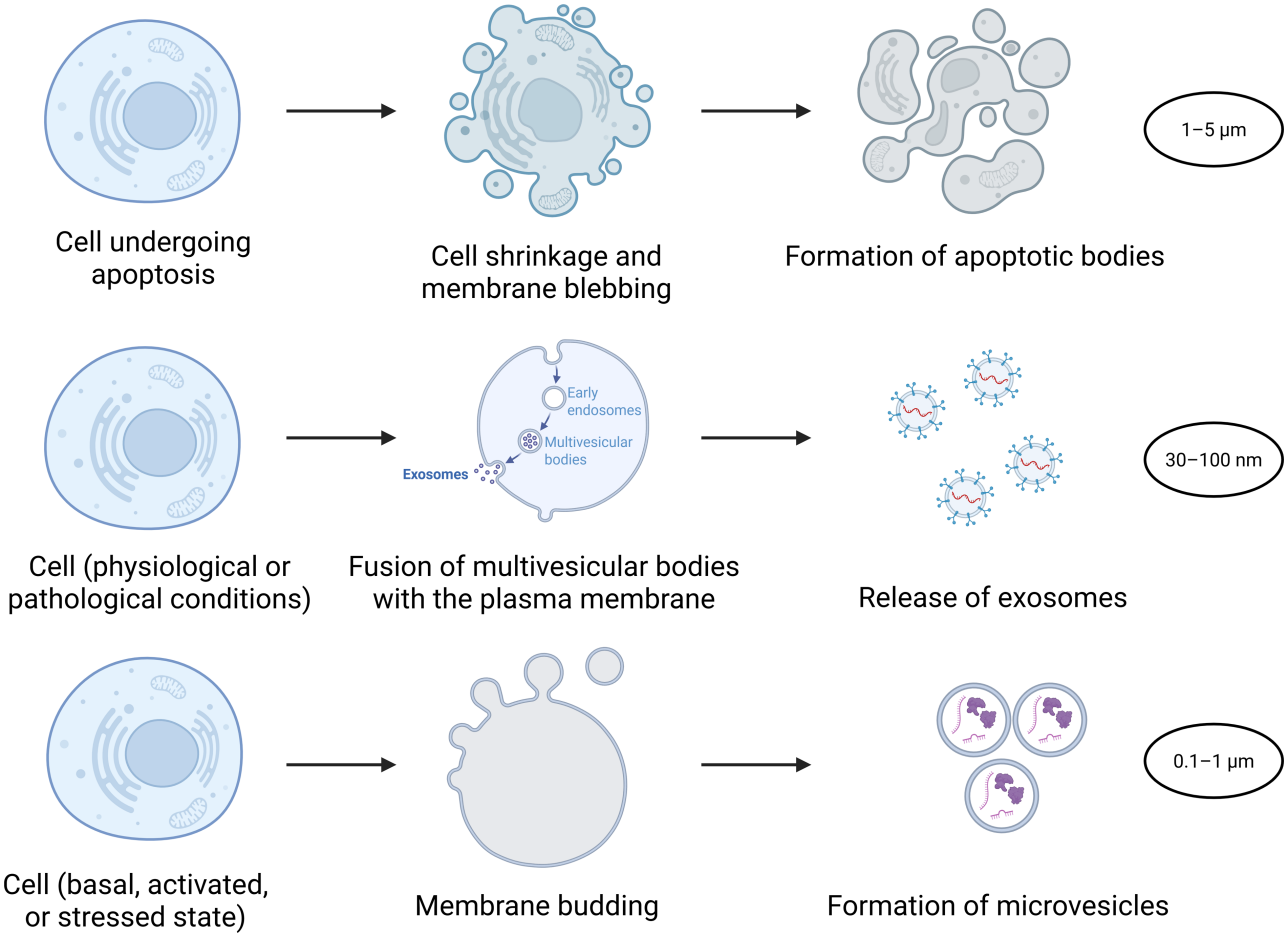
Comparison of cellular processes leading to apoptotic bodies, exosomes, and microvesicles. This figure illustrates three distinct biological pathways by which cells generate extracellular vesicles or cellular fragments. Top row: During apoptosis, the cell undergoes shrinkage and membrane blebbing, ultimately fragmenting into apoptotic bodies. Middle row: exosomes are produced when intracellular multivesicular bodies fuse with the plasma membrane, releasing small vesicles into the extracellular space through exocytosis. Bottom row: microvesicles form through outward budding and fission of the plasma membrane, resulting in larger membrane-bound vesicles containing cytosolic components. These processes differ in their mechanisms of formation, vesicle size, and biological roles in intercellular communication and clearance of cellular debris. Created with BioRender (https://BioRender.com).

Together, these biogenesis pathways contribute to the functional diversity of EVs populations. While classification based on origin remains widely used, it is essential to note that EVs subtypes can overlap in size and composition, reinforcing the need for careful characterization using molecular markers, density gradients, and advanced imaging. Figure 3 compares the cellular processes leading to apoptotic bodies, exosomes, and microvesicles.

EVs are ubiquitous in biological fluids, including blood, saliva, urine, breast milk, cerebrospinal fluid, ascitic and amniotic fluid, underscoring their role in systemic and local cell communication (106). EVs are classified based on their biogenesis, size, and physicochemical properties (107). They are formed by the direct budding of the plasma membrane. Among EVs, exosomes are the best characterized and have garnered significant attention due to their involvement in cellular homeostasis and pathological processes. Exosomes typically exhibit a density of 1.13 to 1.19 g/mL in sucrose gradients (106, 108). However, “small EVs” or “exosomes” are often used based on size, yet there are no universally accepted cut-off values. Moreover, current isolation methods frequently yield heterogeneous EVs populations. Consequently, as most isolation techniques cannot definitively determine EVs origin, the generic term “extracellular vesicles (EVs)” is preferred (44).

EVs’ biogenesis proceeds through several stages. Endocytic vesicles, generated via clathrin-mediated or clathrin-independent endocytosis at the plasma membrane, are trafficked to early endosomes. Subsequently, late endosomes develop from early endosomes via acidification, changes in protein content, and membrane fusion. MVBs arise through the inward budding of the late endosomal membrane, resulting in the accumulation of ILVs. Once formed, MVBs may either fuse with lysosomes, leading to the degradation of vesicle contents, or with the plasma membrane, releasing ILVs into the extracellular space as EVs. This process contributes to membrane turnover and the regulation of surface receptor expression (39, 107).

EVs-associated proteins are classified into two groups: the first group, structural and biogenesis-related proteins, including vinculin, cadherins, CD166, cytoskeletal components such as actin and *β*-tubulin; the second, membrane fusion and trafficking proteins, such as annexins (1–7 and 11) and members of the tetraspanin family (CD9, CD37, CD53, CD63, CD81, CD82) (36).

Beyond their role in intercellular communication, EVs have emerged as active modulators of diverse biological processes. They contribute to immune regulation, angiogenesis, tissue remodeling, and cell survival by transferring functional biomolecules such as cytokines and non-coding RNAs to recipient cells. These properties have positioned EVs as key mediators in regenerative medicine, particularly stem cell-based therapies. In gonadal regeneration, EVs derived from MSCs have shown the ability to replicate many therapeutic effects on their precursor cells, offering a promising cell-free approach. The following section explores the characteristics of MSCs from various tissue sources and the regenerative potential of their EVs in reproductive applications.

## 6 Types of MSCs and their extracellular vesicles

### 6.1 Bone marrow mesenchymal stem cells

BM-MSCs are a diverse group of cells within the bone marrow that provide structural and functional support to hematopoietic cells. First identified by Owen and Friedenstein in 1988 from nucleated bone marrow populations (109), BM-MSCs have been extensively characterized and serve as the benchmark for comparing MSCs from tissue sources (110). These multipotent stem cells can differentiate into several mesenchymal lineages, including adipocytes, osteoblasts, and chondroblasts. Under specific conditions, BM-MSCs can differentiate into non-mesodermal cell types such as endometrial (111), endothelial (112) and granulosa-like cells (113).

In chemotherapy-induced ovarian damage models, BM-MSCs restore ovarian function and prevent gonadal failure by enhancing folliculogenesis (34, 81, 113). For example, Abd-Allah et al. reported that BM-MSCs administered to rats with cyclophosphamide-induced POI improved ovarian architecture, elevated estradiol levels, and reduced expression of Caspase-3, likely due to VEGF-mediated effects (81). Their efficacy is attributed to the secretion of trophic factors such as VEGF and IGF, which promote angiogenesis and ovarian recovery (114, 115).

Beyond ovarian repair, BM-MSCs have also demonstrated the capacity for endometrial repair (32). In Asherman syndrome models, Singh et al. showed that autologous BM-MSCs restored menstruation (116). Additionally, studies have revealed that CD133^+^BM-MSCs can promote endometrial regeneration around the uterine vasculature by secreting IGF-1, thrombospondin 1, and enhancing tissue proliferation (78, 117, 118).

The regenerative effects of BM-MSCs are largely mediated by their EVs. These vesicles are enriched with microRNAs that modulate apoptosis and inflammation. For instance, BM-MSC-derived EVs carrying miR-21 enhance follicular development and reduce GCs apoptosis through the downregulation of PDCD4 and PTEN, and modulation of estradiol and FSH levels (119), which is relevant since GCs play a critical role in nurturing oocytes through hormonal and metabolic support; their loss can severely impair oocyte development (120). Similarly, EV miR-644-5p inhibits GCs apoptosis by targeting the p53 pathway, while miR-144-5p modulates PTEN expression to improve rat ovarian function after the chemotherapy-induced POI model (74, 76).

In males, BM-MSCs and Sertoli cells share common embryonic origins and exhibit overlapping differentiation potential and immunomodulatory functions. Thus, Sertoli cells in the testes play a crucial role in supporting self-renewal, from the stemness stage of spermatogonia to complete spermatogenesis. Administration of BM-MSCs into the testes increased spermatogonia cell counts and repaired the testicular microenvironment in sterile rats (121–123). Moreover, BM-MSCs were able to suppress immune responses to germ cell damage in busulfan (BF)-treated mice (124, 125). Additionally, when BM-MSCs were integrated into an air-liquid interface coculture system, they supported the survival and differentiation of spermatogonial stem/progenitor cells (SSPCs) from prepubertal mouse testes over a 42-day period. BM-MSCs expanded SALL4(+)/OCT4(+) SSPCs, increased VASA(+) germ cells, and promoted the appearance of SCP3(+) spermatocytes and spermatids, thus enhancing maturation and proliferation (126).

BM-MSCs-derived EVs can protect TM3 Leydig cells in cyclophosphamide-induced injury, through the increased expression of StAR, a key protein involved in steroidogenesis, partially restoring testicular endocrine function (127). Moreover, EVs were proven to promote autophagy and enhance survival in Leydig cells through the activation of the AMPK-mammalian target of rapamycin (mTOR) signaling pathway (128). Taher et al. proved that small EVs derived from nuclear factor erythroid 2-related factor 2 (Nrf2)-activated BM-MSCs significantly reduce doxorubicin (DOX)-induced testicular damage and infertility in mice. The activation of the Nrf2 antioxidant pathway in BM-MSCs using Bardoxolone methyl (BaMet) enhanced the antioxidant content of EVs, improving their therapeutic efficacy by increasing testosterone levels, improving testicular architecture, sperm quality, and fertility outcomes (129).

### 6.2 Adipose tissue mesenchymal stem cells

AT-MSCs represent a relatively recent source of MSCs with proven regenerative potential (130–133). Compared to BM-MSCs, AT-MSCs present several practical advantages, including easier harvesting, higher cell yield, and robust immunomodulatory properties. Functionally, AT-MSCs retain the ability to differentiate not only into classical mesenchymal lineages such as adipocytes, chondroblasts, and osteoblasts but also into other specialized cell types, including neural cells, cardiac myocytes, skeletal muscle cells, vascular endothelial cells, and hepatocytes. This extensive differentiation potential corroborates their broad applicability in regenerative medicine (134, 135).

In models of POI induced by cyclophosphamide, AT-MSCs contributed to improving follicle formation, enhancing ovulation, and reducing GCs apoptosis (114). In another study, AT-MSCs transplantation led to improvements in ovarian structure and function, as demonstrated by elevated follicle numbers and neovascularization in treated mice (136). Experimental evidence has shown that AT-MSCs can be used to enhance vascularization by increasing VEGF expression, thereby supporting angiogenesis and improving the viability of ovarian grafts in rat models (137). Their therapeutic efficacy is enhanced when combined with a collagen scaffold, which prolongs cell retention in the ovarian environment of rats with premature ovarian failure, compared to administration of AT-MSCs alone (138, 139).

Despite their therapeutic benefits, caution is warranted. For example, *in vitro* studies using AT-MSCs from women with endometriosis suggest these cells may promote ectopic endometrial cell proliferation, potentially exacerbating disease pathology (57, 140). On the other hand, a study utilizing a combination of AT-MSCs and estrogen demonstrated improved endometrial repair in a rat model of Asherman syndrome, suggesting synergy between hormonal support and stem cell-based therapy for uterine regeneration (141).

Miranda et al. demonstrated the beneficial effects of the conditioned medium of bovine AT-MSCs in a co-culture system with GCs for *in vitro* bovine embryo production, also inducing the upregulation of G6PDH and POU5F1, a key transcription factor associated with pluripotency maintenance in mammals (142, 143). Using the same strategy, the conditioned medium derived from human AT-MSCs exerts anti-cancer effects on ovarian cancer cells. The EVs inhibited proliferation, wound healing, and colony formation in A2780 and SKOV-3 cells by inducing apoptosis through the mitochondrial pathway (144).

In a testicular ischemia–reperfusion injury rat model, Simsek et al. showed that AT-MSCs-derived EVs preserved seminiferous tubule architecture and reduced apoptotic cell death (145). Also, in a rat model of non-obstructive azoospermia (NOA), AT-MSCs-derived EVs demonstrated superior regenerative effects compared to platelet-rich plasma (PRP) by increasing testicular size, restoring testosterone levels, enhancing antioxidant enzymes glutathione peroxidase (GPX), catalase (CAT), and increasing sperm motility and key spermatogenic proteins DAZL and DDX4 (146). However, studies to evaluate the regenerative potential of AT-MSCs-derived EVs in other tissues suggest that they may not fully replicate the regenerative efficacy of living AT-MSCs (147, 148). Underscoring the importance of the appropriate selection of the MSCs-derived-EVs.

### 6.3 Umbilical cord mesenchymal stem cells

UC-MSCs are a highly accessible and abundant source of MSCs known for expressing stem cell-associated markers and differentiating into different mesodermal lineages. These cells display advantages like rapid proliferation, minimal tumorigenic risk, low immunogenicity, and the benefit of non-invasive collection methods (149). Their low immunogenicity is due mainly to minimal expression of major histocompatibility complex (MHC) class I and II molecules, which reduces the probability of immune rejection in allogeneic transplantation (150–152).

Preclinical and clinical studies have shown that UC-MSCs may enhance ovarian activity, either by improving the endocrine function, activating primordial follicles, and reducing cellular apoptosis in POI models, when administered in combination with collagen scaffolds (153, 154). The mechanisms underlying UC-MSCs-mediated ovarian protection involve several signaling cascades. Evidence suggests that UC-MSCs help to mitigate GCs apoptosis by modulating pathways such as mitogen-activated protein kinase (MAPK), G protein-coupled receptor (GPCR), and insulin-related signaling (153, 154). UC-MSCs also secrete pro-angiogenic and trophic factors, including VEGF, HGF, and TGF-β1, further contributing to ovarian recovery and tissue repair (155).

Elfayomy et al. demonstrated that UC-MSCs attenuate apoptosis in ovarian tissue by downregulating molecules involved in Caspase-3-mediated cell death (156). To enhance therapeutic efficacy, UC-MSCs have been integrated into biomaterial scaffolds. For instance, UC-MSCs loaded onto collagen scaffolds can stimulate activation of primordial follicles through phosphorylation of transcription factors such as Forkhead box protein O1 (FOXO1) and FOXO3a, which are key regulators of ovarian development and cell survival (155, 157).

UC-MSCs may also be beneficial in managing PCOS induced by dehydroepiandrosterone (DHEA), as restored hormonal balance and improved ovarian function. These benefits were linked to the suppression of pro-inflammatory cytokines, including IL-1β, TNF-𝛼, and IFN-𝛾, alongside a reduction in the expression of fibrosis-related genes such as connective tissue growth factor (CTGF) (55).

The potential of UC-MSCs in endometrial repair by promoting tissue regeneration at cesarean section scar sites was evaluated (158), and its potential to differentiate into endometrial cell types (159). Their regenerative effect is thought to be mediated through the inhibition of inflammation, attenuation of fibrosis, and enhanced cellular proliferation (160).

miRNAs encapsulated within UC-MSCs-derived EVs play pivotal roles. For example, EVs carrying miR-145-5p have been shown to reduce apoptosis in GCs (161). Additionally, EVs engineered to overexpress therapeutic hsa_circ_0002021 enhanced anti-aging and anti-apoptotic effects in POI models (162). UC-MSCs delivered via collagen-based scaffold further enhanced endometrial regeneration, partly by inducing matrix metalloproteinase 9 (MMP9) expression (163, 164). Also, UC-MSCs have been investigated on trophoblast behavior, revealing that these cells enhance trophoblast migration and proliferation, suggesting a protective role in placental development and function (165). Notably, EVs derived from UC-MSCs have been shown to improve placental structure and angiogenesis in a dose-dependent manner in preeclampsia models, reinforcing the potential for cell-free therapies in managing placental dysfunction (166).

Ding et al. demonstrated that human UC-MSCs-EVs restore ovarian function in a mouse POI model, promoting the proliferation of human GCs and ovarian cells by delivering miR-17-5p, which suppresses SIRT7 expression, thereby downregulating damage markers PARP1, γH2AX, and XRCC6 (167). In a recent study, UC-MSCs-derived EVs improved ovarian morphology, increased follicle counts, and enhanced fertility in *in vivo* POI models, while promoting GCs proliferation and reducing apoptosis *in vitro*. miR-20 b-5p was identified as a key regulator contributing to reduced PTEN expression and activation of the PI3K-Akt pathway, a central mechanism in ovarian recovery (168).

Human UC-MSCs-EVs were successfully tested for fertility preservation after chemotherapy-induced testicular dysfunction model *in vitro* using Sertoli Cells. EVs preserved the spermatogonial stem cell niche, enhanced cell proliferation, and improved hormonal balance in a dose-dependent manner (169).

### 6.4 Amniotic membrane mesenchymal stem cells

Mesenchymal stem cells derived from amniotic membrane (AM-MSCs) can be easily isolated from the amniotic membrane, offering a pragmatic source of cell-based therapies. AM-MSCs exert protective effects in rodent models of POI. Specifically, AM-MSCs administration was associated with reduced GCs apoptosis, enhanced cellular proliferation, and increased angiogenesis; these effects are attributed to paracrine-mediated signaling within the ovarian microenvironment (170, 171). Additionally, AM-MSCs exhibit anti-inflammatory properties. Notably, applying low-intensity pulsed ultrasound (LIPUS) before AM-MSCs transplantation has been shown to enhance therapeutic efficacy (170).

Human AM-MSCs restored spermatogenesis and protected fertility in a mouse model of BF-induced testicular toxicity. Their transplantation improved testicular morphology, increased testosterone levels, and enhanced semen quality by reducing oxidative stress, apoptosis, and upregulating germ cell-specific DAZL, DDX4, Miwi, and meiosis-related SCP3, Cyclin A1, Stra8 genes and proteins (172).

The conditioned medium of human AM-MSCs mitigates testicular failure. In a rat POI model induced by ionizing radiation, whether via scrotal or whole-body irradiation, both cell transplantation and conditioned medium administration restored fertility. In males, this intervention increased seminiferous tubule diameter and epithelial height, along with improved levels of FSH, LH, and testosterone. In females, FSH and LH were increased. Mechanistically, endoplasmic reticulum stress and apoptosis were suppressed by downregulating GRP78, IRE1𝛂, CHOP, Caspase-12, Caspase-3, and reducing TUNEL-positive cells (173, 174).

### 6.5 Placenta-derived mesenchymal stem cells

Pc-MSCs are obtained by dissecting the chorionic villi or chorionic plate, regions of the placenta (175). They present low immunogenicity, making them suitable for allogeneic transplantation. Compared to other MSCs, Pc-MSCs exhibit enhanced proliferative and differentiation capacities, which are helpful in tissue repair and immune modulation (114).

In a murine POI model, Pc-MSCs contributed to the repair of ovarian function by regulating cytokine networks. These cells modulated circulating estradiol and FSH levels toward more physiological values and upregulated anti-Müllerian hormone (AMH). AMH is a biomarker of ovarian reserve and follicular activation. The therapeutic actions of Pc-MSCs in this context involve activation of the PI3K/Akt signaling pathway, which is known to regulate cellular survival and proliferation (176–178).

Li et al. showed that Pc-MSCs suppressed GC apoptosis by downregulating the IRE1-mediated endoplasmic reticulum stress pathway, further preserving ovarian morphology and function in chemotherapy-induced POI models (77). Additional evidence from the ovariectomized rat model supports that Pc-MSC transplantation can stimulate estrogen production and induce the expression of genes associated with folliculogenesis, contributing to partial ovarian regeneration (179).

Beyond ovarian restoration, Pc-MSCs are promising in addressing preeclampsia, a hypertensive disorder specific to pregnancy. Pc-MSCs secrete growth factors and immunoregulatory cytokines, which are believed to influence key signaling pathways involved in preeclampsia development. Moreover, certain microRNAs, like miR-222, have been found to modulate the behavior of Pc-MSCs by targeting genes such as BCL2L11, a regulator of apoptosis, thereby affecting cell survival and differentiation in preeclamptic environments (180). Pc-MSCs can interact with cell cycle regulatory pathways, specifically those governing the G1/S phase transition, in preeclamptic placental tissue, which may contribute to their therapeutic effects (181).

Recently, Le et al. assessed the secretome of estrogen receptor (ER)positive human Pc-MSCs (ER^+^Pc-MSCs), particularly when primed with estradiol (E2-conditioned medium), which holds strong therapeutic potential for gonadal regeneration in a mouse POI model. *In vivo* and *in vitro* experiments demonstrated that treatment with conditioned medium and with E2-conditioned medium derived from ER^+^Pc-MSCs responds to the ovarian estrogen niche via ER𝛂 expression, effectively restoring ovarian morphology and function. This secretome promoted folliculogenesis, steroid biosynthesis, and GCs survival, contributing to the recovery of ovarian function. They also modulated genes involved in the ovarian circadian clock, which plays a key role in regulating estrogen biosynthesis. Mechanistically, the regenerative effects were mediated by angiogenin and EVs miRNAs targeting apoptotic genes such as Caspase-3 and BIM, POI rescue such as PTEN and PDCD4, estrogen synthesis (CYP19A1), ovarian clock regulation (E4BP4, REV-ERB𝛂), and fibrosis (COL1A1) (182). In a co-culture system with Pc-MSCs-derived EVs, miR-139-5p lowered apoptosis and GCs damage induced by cisplatin *in vitro* (183).

### 6.6 Spermatogonia stem cells and oogonial stem cells

Although the evidence for EV-mediated communication from SSCs, oogonial stem cells (OSCs), and PSCs is still preliminary, these cell types represent emerging and rapidly evolving areas of interest in gonadal regeneration. Their inclusion here is intended to highlight future therapeutic directions rather than established EV-based pathways. SSCs and OSCs are essential for direct gametogenesis and fertility restoration. SSCs in the testes are critical for maintaining continuous spermatogenesis and play a crucial role in male fertility. Preclinical studies have demonstrated successful restoration of spermatogenesis and functional sperm production following SSC transplantation, especially relevant for prepubertal cancer patients undergoing gonadotoxic therapies (184–186). Recent studies have shown that SSCs can release EVs, potentially contributing to the regulation of the germ cell niche. MVBs, precursors to EVs, have been identified in type A spermatogonia, and a dense population of EVs has been localized near the basement membrane of seminiferous tubules across various mammalian species (187). In murine models, undifferentiated spermatogonia expressing the surface marker Thy1 were shown to secrete EVs that inhibit SSC proliferation, suggesting a role in maintaining homeostasis within the testicular microenvironment. Transmission electron microscopy (TEM) analysis demonstrated that most (83%) of testicular EVs interacted with spermatogonia, whereas only 17% contacted Sertoli cells. However, in this study, EVs were isolated from enzymatically digested testicular tissue, a procedure that may influence EV secretion and alter their cargo (187).

Yun et al. isolated EVs from the C18-4 SSC cell line and found that these EVs enhanced testosterone production in TM3 Leydig cells. This paracrine interaction reveals the ability of SSC-derived EVs to modulate the endocrine function of neighboring somatic cells (188). Together, these studies suggest that SSC-derived EVs participate in bidirectional communication within the testicular niche, thereby regulating the functions of both germ and somatic cells.

The existence and functional role of OSCs in postnatal mammalian ovaries remain controversial. While some studies have reported mitotically active germ cells capable of neo-oogenesis, the physiological relevance and existence of functional OSCs in adult ovaries remain controversial (189). Recent advancements have revealed that OSCs, when seeded into biomimetic ovarian scaffolds, often in combination with MSCs, can differentiate into oocyte-like cells capable of meiotic progression (190, 191). These findings highlight the potential of OSCs for treating female infertility; however, specific EV studies are necessary, and further validation is essential to confirm their efficacy and functional competence (192).

### 6.7 Pluripotent stem cells (iPSCs, ESCs) and *in vitro* gametogenesis

Among the stem cells investigated for gonadal regeneration, embryonic stem cells (ESCs) have demonstrated remarkable potential due to their capacity to differentiate into all germ and somatic cell types. Over the past decade, considerable progress has been made in directing ESCs toward primordial germ cell (PGC)-like cells and, under optimized conditions, toward functional gametes (193, 194). These ESC-derived germline-like cells express key markers such as DAZL, VASA, and OCT4, which are essential for primordial germ cell specification and entry into meiosis (195, 196).

Along with germ cells, ESCs can differentiate into Sertoli-like and Leydig-like cells, particularly when co-cultured with embryonic gonadal somatic cells or exposed to inductive signaling factors (197–199). Thus, ESCs may support both gamete development and the broader endocrine functions of the gonads. Despite advances, achieving full maturation remains difficult. Preserving epigenetic fidelity remains a significant challenge for the clinical translation of epigenetic therapies. The induction of meiosis in ESC-derived cells has been achieved using BMP4 stimulation, reinforcing the potential for *in vitro* gametogenesis (143, 200). The discovery of SOX17 as a key regulator of human PGC fate has further clarified the molecular mechanisms involved in ESC differentiation in reproductive cell lineages (201). These findings offer new insights for fertility restoration therapies and the study of human germ cell development.

Induced pluripotent stem cells (iPSCs) have revolutionized regenerative medicine by enabling the development of patient-specific therapies. These cells, reprogrammed from adult somatic cells using transcription factors such as OCT4, SOX2, KLF4, and c-MYC, exhibit pluripotency, allowing differentiation into various cell types, including gonadal somatic cells and germ cells (143, 202). Recent advances in differentiation protocols demonstrate that iPSCs can generate functional ovarian follicles and granulosa-like cells, critical for hormone production, folliculogenesis, and oocyte maturation, representing a potential breakthrough for patients with POI and other reproductive disorders (202). Similarly, iPSCs have been successfully differentiated into Sertoli-like and Leydig-like cells, highlighting their potential to restore testicular function in cases of male infertility (203–205).

Beyond therapeutic application, iPSC-derived gonadal cells provide robust models for studying human gonadal development, reproductive aging, and endocrine disorders. For example, iPSC-based disease models have elucidated mechanisms underlying conditions such as PCOS, gonadal dysgenesis, hypogonadism, and gonadal cancer, facilitating targeted drug discovery and testing of novel reproductive therapies (206–208). Despite this promise, challenges remain, including maintaining genomic stability during reprogramming, optimizing differentiation protocols to improve efficiency and reproducibility, and mitigating tumorigenic risk (209). Moreover, ethical concerns surrounding genetic modification and artificial gametogenesis require careful regulatory consideration and oversight. Ongoing advances in biomaterial-based culture systems, 3D organoid models, and CRISPR-Cas9-mediated gene editing are helping to overcome these barriers and accelerate the clinical translation of iPSC-based therapies.

A recent study demonstrated that EVs released by iPSC-derived cardiomyocytes from familial dilated cardiomyopathy (DCM) patients promote fibrogenesis in cardiac fibroblasts. These EVs, particularly under angiotensin II stimulation, were enriched in miR-218-5p, which enhanced TGF-*β* signaling by downregulating the inflammation suppressor TNFAIP3. This finding reveals the ability of iPSC-derived EVs to modulate disease-specific molecular signatures and actively modulate fibrotic pathways (210). However, there is a lack of literature exploring the role of iPSC-EVs in reproductive medicine, indicating an unexplored avenue for future research. Table 1 depicts some aspects of the effects of various organs’ MSCs-derived EVs in gonadal regeneration.

**Table 1:** Mesenchymal stem cell-derived EVs in gonadal regeneration.

Stem cell source	EV content	Main effect	Reference
BM-MSCs (rat)	miR-144-5p	Inhibition of GCs apoptosis by targeting PTEN	(74)
	miR-21	Inhibition of GCs apoptosis via PDCD4 and PTEN targeting	(119)
	SOD and CAT (262)	Improved antioxidant activity, testosterone levels, testicular cell populations, sperm viability/motility via the Nrf2 pathway	(129)
	circLRRC8A	Protection against oxidative stress and senescence via the circLRRC8A/miR-125a-3p/NFE2L1 axis	(263)
BM-MSCs (mouse)	miR-644-5p	Inhibition of GCs apoptosis via p53 targeting	(76)
	StAR protein	Protection of TM3 Leydig cells via increased StAR expression	(127)
	N/A	Reduction of spermatogonia cell death, promotion of Leydig cells’ autophagy via AMPK-mTOR	(128)
	N/A	Upregulation of MRP1/BCRP1, enhanced GCs proliferation, reduced cleaved Caspase-3 expression	(264)
	miR-126	Enhanced endothelial proliferation, migration, angiogenesis via PI3K/Akt/eNOS and VEGF/FGF	(65)
BM-MSCs (human)	N/A	Restored estrous cycle and hormone levels	(265)
	N/A	Reduced senescence and apoptosis in GCs	(266)
	PDGF, EGF, FGF	Induced angiogenesis via NF-kB; increased endothelial expression and angiogenic factors	(267)
AT-MSCs (rats)	Multiple miRNAs	Attenuated oxidative stress/inflammatory response, promoted spermatogenic cell proliferation via PI3K/Akt and MAPK/ERK1/2	(268)
AT-MSCs (human)	miR-323-3p	Promoted GCs proliferation, inhibited apoptosis via PDCD4 targeting	(269)
AM-MSCs (human)	miR-320a	Reduced ROS in GCs and oocytes by regulating SIRT4	(270)
Pc-MSCs (human)	miR-222	Increased PC-MSCs apoptosis in preeclampsia by targeting BCL2L11	(180)
	miR-139-5p	Induced GCs proliferation via PTEN targeting	(183)
	N/A	Reduced senescence and apoptosis in SVOG cells	(266)
UC-MSCs (human)	SOD and GPX (271)	Inhibited ferroptosis and GCs apoptosis via the Nrf2/GPX4 pathway	(272)
	miR-29a	Improved ovarian function via Wnt/β-catenin activation by targeting HBP1	(273)
	N/A	Promoted GCs proliferation and reduced apoptosis via PI3K/Akt	(274)
	miR-126-3p	Reduced GCs apoptosis, promoted angiogenesis via SPRED-1 and PIK3R2 (275)	(66)
	miR-22-3p	Reduced GCs apoptosis via the KLF6 and ATF4-ATF3-CHOP pathway targeting	(276)
	miR-145-5p	Reduced GCs oxidative injury and endoplasmic reticulum stress by modulating XBP1	(161)
	hsa_circ_0002021	Enhanced anti-aging, reduced GCs senescence via CDK6 regulation	(162)
	N/A	Promoted placental angiogenesis via VEGF, sFlt1 suppression	(166)
	miR-17-5p	Promoted GCs proliferation via SIRT7 targeting	(167)
	miR-20b-5p	Promoted GCs proliferation and reduced apoptosis, reduced PTEN, and activated PI3K/Akt	(168)
	N/A	Preserved spermatogonial stem cell niche and improved Sertoli cells proliferation	(169)
	N/A	Decreased FSH and GCs apoptosis; increased estradiol and AMH	(277)
	N/A	Promoted GCs proliferation and reduced apoptosis in rat follicles	(277)
	circBRCA1	Improved mitochondrial function and reduced senescence via miR-642a-5p/FOXO1	(278)
	HGF	Activated primordial follicle via PI3K-Akt pathway	(279)
	N/A	Improved ovarian functions and GCs proliferation via the Hippo pathway	(280)
	miR-21	Promoted estrogen production in ovarian GCs via LATS1-mediated phosphorylation of LOXL2 and YAP	(281)
SSCs (mouse)	N/A	Suppressed the SSCs’ proliferation	(187)
	N/A	Enhanced testosterone levels in TM3 Leydig cells	(188)

## 7 EVs from other sources for gonadal regeneration

### 7.1 EVs’ impact on ovarian follicle

EVs isolated from human follicular fluid closely resemble the donor’s plasma profile, enabling the identification of miRNAs specifically derived from ovarian follicular cells rather than systemic circulation. Bioinformatic analyses suggested that these miRNAs play critical roles within the follicular environment. For instance, miR-99a, miR-100, miR-132, and miR-218 may be linked to follicle maturation, while miR-132, miR-212, and miR-214 may influence meiotic resumption by suppressing genes that inhibit follicular development (211).

The miRNAs from both human and animal follicular fluids have been identified in EVs. Silveira et al. documented the presence of miRNAs and protein-loaded EVs in equine follicular fluid (212, 213). Then, Sohel et al. identified EVs in bovine follicular fluid (214). Both showed that miRNA profiles modulate oocyte growth, and recently, it was observed that miRNAs promote cumulus cell expansion (215). Accumulating evidence implicates EV-derived miRNAs in the regulation of folliculogenesis and in the pathophysiology of reproductive disorders (216–219). For example, miR-132 and miR-320, which influence steroid production in KGN cells, therefore, participate in PCOS (220). Beyond miRNAs, EVs cargo includes macromolecules such as mRNAs, long non-coding RNAs, mitochondrial DNA, and proteins, expanding their potential regulatory roles (39, 40, 107, 221).

Although human oocytes can synthesize various classes of small non-coding RNAs, including piRNAs, miRNAs, and endo-siRNAs, their specific functions in oogenesis and follicular development remain unresolved (222). Besides, proteomic analyses revealed that EVs conserved proteins reflective of their cellular origin (40). Notably, many of these proteins/cytokines lack a classical signal peptide for secretion, suggesting EV-mediated transport as an alternative route (223). EVs may also deliver signaling ligands such as Wnts, which can activate Frizzled receptors in a stage-specific manner during follicular development (224). Finally, oocytes may also secrete EVs, whose content, like miR-371, would be relevant to pluripotency and cellular reprogramming.

**Figure 4:**
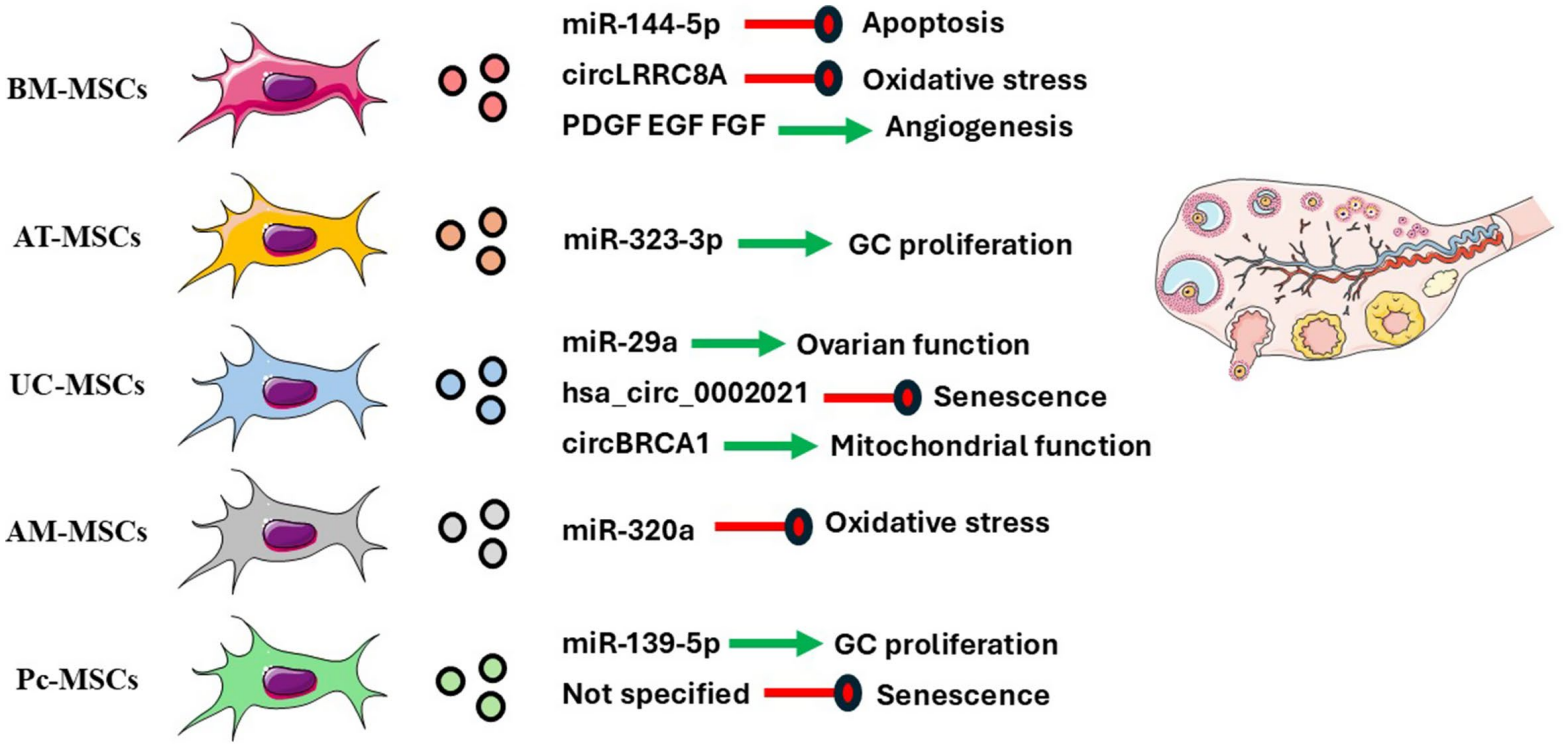
Molecular factors carried by mesenchymal stem cells (MSCs)-derived extracellular vesicles (EVs) from different tissue sources and their reported effects on ovarian cells and function. BM-MSCs (bone marrow), AT-MSCs (adipose tissue), UC-MSCs (umbilical cord), AM-MSCs (amniotic membrane), and Pc-MSCs (placental) release EVs enriched in distinct microRNAs, circular RNAs, and growth factors. These cargo molecules modulate key ovarian processes, including apoptosis, oxidative stress response, angiogenesis, granulosa cell (GCs) proliferation, ovarian function, and cellular senescence, as indicated by the colored arrows. Green arrows indicate beneficial or stimulatory effects, while red arrows denote inhibitory effects. Created using elements from Servier Medical Art (https://smart.servier.com/); licensed under CC BY 4.0.

A central question in EV biology is whether they serve solely as passive cargo carriers or exert active signaling functions (225). Evidence suggests that proteins like *β*-catenin-regulated by tetraspanins CD9 and CD82, can suppress Wnt signaling by reducing β-catenin levels (226). In this context, EVs enriched in miR-132, miR-212, and miR-214, known to target PTEN, may activate the PI3K/Akt pathway, a central regulator of follicle activation, recruitment, and ovulation (211, 227, 228). Figure 4 illustrates the effects of EVs from multiple sources in ovarian development.

### 7.2 EVs’ impact on testicular development

The surrounding niche regulates the maintenance and differentiation of SSCs, which are essential for male reproductive potential. EV-derived miR-486-5p from Sertoli cells has been shown to suppress PTEN expression while increasing Stra8 levels, thereby promoting SSC maturation (229). miRNAs have been identified as regulators of SSC renewal (230), differentiation (231), and even sperm viability and fertilization capacity.

Spermatogonia serve as the progenitors of sperm cells through their mitotic activity. BM-MSCs-derived EVs support their differentiation into germ cells (232). EVs released near the basal membrane of seminiferous tubules appear to modulate SSC proliferation (233, 234). Given that spermatogonial density correlates strongly with sperm quality and fertility, EV-mediated modulation of SSC behavior holds therapeutic outcomes (233, 234). *In vivo* applications, especially EV-based drug delivery systems, are being assessed to understand if EVs can restore spermatogenesis and sperm functions (235, 236).

In male germ cells, loss of Drosha impairs miRNA expression, leading to defective meiosis and reduced testis weight. In the rodent model of testicular toxicity, miR-423-5p and miR-128-3p emerged as biomarkers of damage, as detected through transcriptomic profiling (237). This highlights the importance of miRNA regulation, which may also be mediated via EV delivery in the testicular niche.

EVs derived from MSC, irrespective of their source, have demonstrated immunomodulatory, angiogenic, and anti-inflammatory effects (238, 239). Their capacity to reduce oxidative stress makes EVs a valuable tool for improving sperm quality. In preclinical studies, EVs from amniotic fluid improved sperm motility, concentration, and spermatogonial populations (232). Moreover, MSCs-derived EVs have protective effects during sperm cryopreservation, enhancing cell viability and membrane integrity, partly attributed to adhesion molecules like CD44 and CD29 (240, 241).

**Figure 5:**
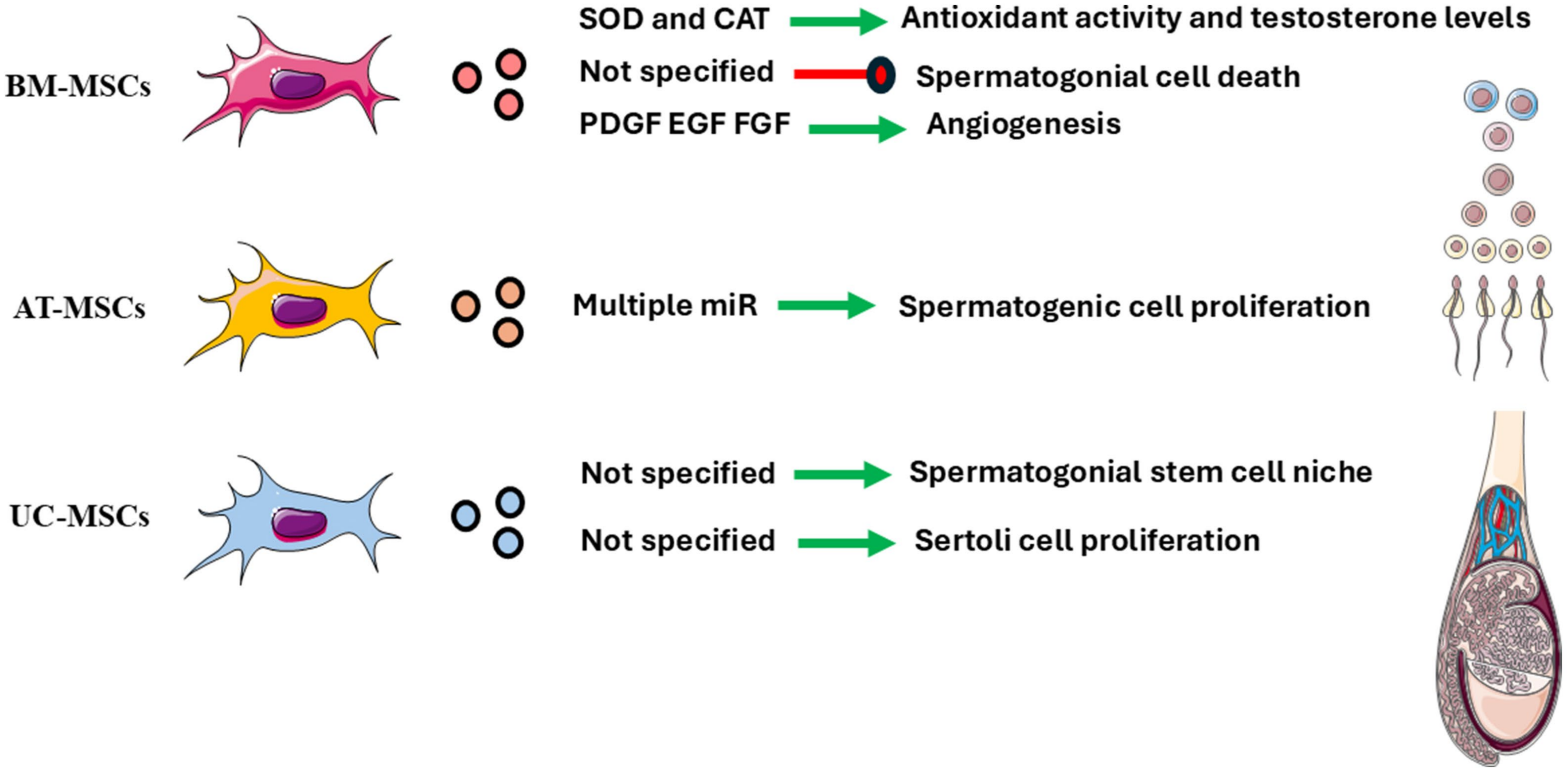
Key bioactive components identified in extracellular vesicles (EVs) derived from different mesenchymal stem cell (MSC) sources and their reported effects on testicular cells and function. EVs from BM-MSCs (bone marrow), AT-MSCs (adipose tissue) and UC-MSCs (umbilical cord) contain distinct molecular cargos—including miRNAs, antioxidant enzymes, and growth factors—that influence antioxidant activity, testosterone production, spermatogonial and spermatogenic cell proliferation, Sertoli cell support, angiogenesis, and apoptosis. Green arrows indicate beneficial or stimulatory effects, while red arrows denote inhibitory effects. Created using elements from Servier Medical Art (https://smart.servier.com/); licensed under CC BY 4.0.

Sertoli cells orchestrate male germ cell development by providing trophic support. They regulate spermatogenic progression via EV secretion for regenerative purposes (242). Epigenetic mechanisms regulate Sertoli cell function, with Dicer deficiency leading to altered miRNA levels and disrupted expression of key regulators, including GDNF, GATA1, WT1, and SOX9. However, the full scope of EV contributions to testicular development and regeneration remains incompletely understood. Figure 5 illustrates the effects of EVs from multiple sources on testicular development.

## 8 Use in organoids and 3D culture systems

Gonadal organoids, derived from human pluripotent stem cells (hPSCs), have emerged as powerful tools for modeling early gonadal development and *in vitro* gametogenesis. These three-dimensional structures closely recapitulate gonadal architecture and function, providing a controlled platform to investigate the mechanisms underlying germ cell development and differentiation (243, 244). Recent advancements in organoid culture platforms, including bioreactor-based systems and scaffold-free aggregation techniques, have enabled the generation of testicular and ovarian organoids that express stage-specific markers, organize into compartmentalized structures, and even initiate meiotic progression (245, 246). These organoids exhibit a cellular organization resembling fetal testis development, expressing key markers such as SOX9, AMH, and GATA4, which are essential for Sertoli cell function and seminiferous cord formation (88). Moreover, Leydig-like cells within these organoids express CYP17A1 and INSL3, indicating their potential to produce testosterone (203). Transcriptomic analyses have shown that these hPSC-derived testicular organoids closely resemble human fetal gonads at 6–8 weeks of gestation, suggesting their potential for studying early-stage testicular differentiation and androgen biosynthesis (247–249). Furthermore, a novel 3D organoid culture system has been developed to induce the formation of oocytes from E11.5 premeiotic female germ cells, demonstrating the potential of ovarian organoids to replicate the microenvironment for oocyte maturation (250). These findings support their application in studying early-stage testicular differentiation, androgen biosynthesis, and male germline development.

Although organoids capture the structural complexity of gonads, they often lack maturation and vascular integration. This limits their long-term utility in clinical settings. Protocols for guiding murine and human pluripotent stem cells toward gonadal lineages have undergone significant evolution, enabling the *in vitro* generation of progenitors that resemble early developmental stages. Transcriptomic profiling of *in vitro*-derived murine gonadal cells has shown substantial similarity to embryonic day 11.5 (E11.5) progenitors, a critical window in sex determination and the establishment of germ cell niches.

In human models, Sertoli-like cells differentiated from 46, XY hiPSCs have demonstrated the ability to express testis-specific markers, secrete AMH, exhibit directed migration, and self-organize into tubular structures, mimicking early testicular cord formation (208). These findings confirm the fidelity of *in vitro* differentiation and validate organoids as platforms for reconstructing developmental architecture. Notably, hiPSCs derived from patients with 48, XY disorders of sex development (DSDs), particularly those carrying NR5A1 (SF-1) variants, fail to form structured tubules and exhibit disrupted gene expression profiles, mirroring aspects of the disease phenotype. This highlights the potential of gonadal organoids as disease modeling platforms, capable of capturing genotype–phenotype correlations and providing insight into the molecular basis of sex development disorders (247).

The development of testicular organoids has opened up new avenues for *in vitro* modeling of spermatogenesis, providing a controlled and scalable platform for investigating male fertility and germ cell development. These organoids self-organize into tubule-like structures that recapitulate key features of testicular architecture, including compartmentalization between Sertoli and germ cells, a prerequisite for functional spermatogenesis (245, 247, 251).

Transcriptomic and immunohistochemical analyses have confirmed that these organoids express stage-specific markers associated with germ cell maturation, and in some models, meiosis has been initiated. Such systems not only provide insight into the dynamics of human germ cell development but also enable the evaluation of toxicological agents, genetic mutations, and culture conditions that influence SSC behavior (252, 253). Although complete spermatogenesis culminating in mature spermatozoa remains elusive, the capacity to mimic early and intermediate stages positions testicular organoids as a promising tool for future fertility restoration strategies, particularly for prepubertal boys undergoing gonadotoxic therapies.

A microfluidic testis-on-chip platform incorporating BM-MSCs has been developed to support long-term *in vitro* spermatogenesis in prepubertal mouse testes. This demonstrates that this system provides continuous flow and sustained paracrine signaling. Over 42 days, maintained SALL4(+) and PLZF(+) spermatogonial stem/progenitor cells, promoted differentiation into c-Kit(+) spermatogonia, VASA(+) germ cells, and spermatids, enhancing testosterone production and tubular growth (254). These findings underscore the significance of organoid and microfluidic technologies as preclinical platforms for modeling gonadal biology, evaluating regenerative therapies, and ultimately supporting therapeutic efforts in patients affected by gonadotoxic insults or congenital defects.

Despite growing interest in organoids and 3D culture systems for modeling reproductive biology, research on EVs secreted specifically by reproductive organoids remains limited. Preliminary observations from a bovine oviductal organoid model have confirmed the release of EVs; however, their cargo, mechanisms of biogenesis, and functional relevance were not explored (255). This highlights a critical gap in our understanding of how physiologically relevant organoids can be utilized to investigate EV-mediated signaling in the regeneration of gonadal and reproductive tissues. Such research could provide novel insight into cell–cell communication in reproductive tissues, enhance the fidelity of *in vitro* models, and pave the way for EV-based diagnostic and therapeutic applications in reproductive medicine.

## 9 Conclusion

MSCs-derived EVs represent a promising and versatile tool in reproductive regenerative medicine. Accumulating preclinical evidence suggests that EVs can mimic the functions of their precursor MSCs through various mechanisms, including the inhibition of apoptosis, stimulation of angiogenesis, immunomodulation, and suppression of fibrosis. These nanoscale, cell-free carriers deliver functional biomolecules such as microRNAs and proteins that directly modulate the behavior of target cells within the gonadal microenvironment.

In ovarian failure models, EVs have been shown to restore folliculogenesis, enhance oocyte quality, and normalize hormonal profiles. In testicular models, they contribute to the recovery of spermatogenesis, improve Sertoli and Leydig cell function, and reduce germ cell apoptosis. In addition to their regenerative potential, EVs exert potent anti-inflammatory and antifibrotic effects, reestablishing a supportive gonadal niche for tissue repair and functional recovery.

Despite this advance, EV-based therapies face critical translational challenges. The lack of standardized protocols for EV isolation and characterization, as well as the absence of established regulatory frameworks, pose significant barriers to clinical application. In conclusion, MSCs-derived EVs offer a paradigm shift in reproductive medicine, from symptom management toward biological restoration of gonadal function. EVs hold the potential to become the heart of next-generation cell-free therapies.

EV biogenesis itself offers a powerful platform for loading therapeutic cargoes relevant to gonadal regeneration (256). Exosomes generated through the endosomal pathway can be engineered for selective incorporation of small RNAs or proteins by exploiting ESCRT-dependent mechanisms, tetraspanin-associated sorting, or fusion tags such as Lamp2b and CD63 motifs (257). Similarly, microvesicle budding from the plasma membrane can be modulated through cytoskeletal or lipid-raft signaling to enrich regenerative factors (258). By overexpressing specific miRNAs involved in folliculogenesis, steroidogenesis, or SSC maintenance, donor cells can be programmed to direct these molecules into EVs through endogenous sorting routes (259, 260). Furthermore, manipulating donor-cell stress cues can shift EV composition toward pro-regenerative factors (261). Harnessing these biogenetic pathways provides a biologically compatible means to package therapeutic cargoes—such as miRNA clusters, growth factors, or anti-apoptotic proteins—into EVs capable of targeting and repairing damaged ovarian and testicular tissue.
